# Relationship between *hukou* conversion and psychological integration of rural older migrants: the mediating effect of accessibility to health resources

**DOI:** 10.1186/s12877-024-05110-1

**Published:** 2024-06-04

**Authors:** Tianxin Cai, Shilong Ma, Renyao Zhong, Vivian W. Q. Lou

**Affiliations:** 1 School of Public Administration, East China Normal University, 3663 North Zhongshan Road, Putuo District, Shanghai, China; 2 School of Public Administration and Emergency Management, Jinan University, Guangzhou, China; 3 Department of Social Work and Social Administration, University of Hong Kong, Hong Kong, China

**Keywords:** Rural older migrants, *Hukou* conversion, Accessibility of health resources, Psychological integration, Mediating effect

## Abstract

**Objective:**

This study investigates the relationship between *hukou* conversion and the psychological integration of rural older migrants, exploring the mediating role of accessibility to health resources.

**Methods:**

The 3,963 valid samples of rural older migrants included in the study were sourced from the 2017 China Migrants Dynamic Survey (CMDS). The study established a multiple linear regression model for estimation and utilized inverse probability-weighted regression adjustment (IPWRA) method to correct for the selection bias of *hukou* conversion.

**Results:**

Compared to older migrants with rural *hukou*, merit-based (β = 0.384, 95% CI: 0.265 to 0.504), family-based (β = 0.371, 95% CI: 0.178 to 0.565) and policy-based (β = 0.306, 95% CI: 0.124 to 0.487) converters have significantly higher psychological integration. These findings remain robust even after addressing the potential issue of endogenous selection bias using the IPWRA method. Bootstrap mediating effect tests indicate that *hukou* conversion can indirectly affect psychological integration through the mediator role of health resources accessibility.

**Conclusion:**

Accessibility of health resources mediates the association between *hukou* conversion and psychological integration. Policymakers should enhance the implementation of *hukou* conversion, strengthen the health resource guarantee system, and achieve a deeper psychological integration among rural older migrants.

## Introduction

With the acceleration of urbanization and the trend of population aging, the current Chinese population is undergoing significant changes characterized by mobility, separation, and integration. The emergence of rural older migrants is an inevitable result of the development of new urbanization and social modernization [1]. According to the “2018 China Migrant Population Development Report” published by the former National Health Commission, the scale of older migrant population has grown rapidly since 2000, increasing from 5.03 million in 2000 to 13.04 million in 2015, with an average annual growth rate of 6.6% [2]. As a distinct subset within the older population, rural older migrants’ living conditions and health status represent important issues in the context of the new era, including family eldercare, social integration, and policy reform.

As a significant factor influencing the population structure in aging societies, older migrants have attracted attention from western academia since the 1970s [3]. In the cultural context of China, the migration of rural residents to urban areas is the most prominent characteristic of population mobility. Most of these rural-to-urban migrants face strong institutional, economic, cultural, and social barriers, due mainly to the household registration system—*hukou* [4]. Among them, rural older migrants face unique challenges stemming from the intersection of age-related vulnerabilities and the complexities of adapting to new urban environments [5]. In China, this cohort is more vulnerable due to their relatively poor socio-economic status and accessibility to social welfare, low educational level and differences in lifestyle compared to their urban counterparts [6]. Thus, research on their health outcomes, including mental health and healthcare access, is essential for informing policies and interventions.

Social integration, as a multi-dimensional concept, mainly includes economic, social, cultural, and psychological integration [7]. Among those, psychological integration is defined as the process of undergoing psychological and emotional changes in terms of identification with one’s own social identity and sense of belonging [8], which is widely regarded as the pinnacle and final stage of social integration [9]. Empirical studies have confirmed the association between psychological integration and mental health. Positive psychological integration contributes to preserving the self-esteem of the migrant population [10], enhancing positive self-identity [11], ultimately reducing their psychological distress and promoting their mental well-being [12]. Therefore, for the purpose of maintaining the health of rural older migrants, there is a pressing need to investigate interventions that promote their psychological integration.

According to social identity theory, psychological integration is reflected in an individual’s sense of social identity and belonging [13]. Under the cultural background of China, *hukou* system has long been a fundamental aspect of the social structure, profoundly shaping citizen’s social identities [14]. The *hukou* system historically served as a means of controlling internal migration. It was designed to control population mobility and promote the development of heavy industry in cities, helping the government manage resources and maintain social stability [15, 16]. However, it also classified individuals into either rural or urban *hukou* categories, creating the urban-rural dichotomy that significantly influences access to social services, education, health resources and employment opportunities [17]. Since the *hukou* system is witnessed as the main institutional obstacle to the social integration of migrants [18], it has undergone reforms aiming to promote citizenization of rural migrants in recent years.

In 2014, China initiated the new-type urbanization. On July 24, 2014, the State Council issued *the Opinions of the State Council on Further Promoting Hukou System Reform* [19], explicitly pointing out that the distinctions between rural and urban *hukou* should be eliminated and a unified urban and rural *hukou* system should be established. Under the policy of relaxing hukou restrictions, numerous migrants have experienced a process of *hukou* conversion (i.e., transferring rural *hukou* to urban *hukou*) mainly through two paths: one is through self-effort, typically accessible to migrants with higher levels of education, skills, and financial resources, while the other is driven by policy changes that incorporate rural residents’ hometowns into urban areas [20].

The greatest benefit of transferring one’s *hukou* is access to citizenship rights, including local-urban welfare rights, economic opportunity, and social status [21]. Previous research has demonstrated that *hukou* converters in urban areas have a clear and significant advantage in healthcare access than their counterparts who have not converted their *hukou* type [22]. Moreover, the health benefits from *hukou* conversion have been confirmed. Rural migrants who obtain urban *hukou* are reported to have better health conditions compared to their rural *hukou* counterparts [23]. Regarding different types of *hukou* conversion, policy-based converters generally exhibit better psychological health than family- and merit-based converters who are selected on individual traits [24]. Furthermore, the positive association between the accessibility of health resources and psychological well-being has been theoretically and empirically confirmed [25]. A recent study found that health rights accessibility significantly enhances the psychological integration among minority rural migrants [26]. Therefore, the accessibility of health resources might mediate the association between *hukou* conversion and psychological integration.

Although existing studies have gained fruitful results, they have the following limitations. First, since previous literature mainly focused on the effect of *hukou* status (rural vs. urban), it lacks exploration of the *hukou* conversion (rural-to-urban vs. rural) in the context of Chinese *hukou* reforms. Second, previous studies have mainly focused on the psychological well-being of migrant adolescents and workers. Little attention has been paid to the rural older migrants, despite their status as a more vulnerable group. Third, while numerous studies have explored multiple dimensions of psychological well-being, few have specifically focused on the psychological integration, which is a crucial element to the overall psychological well-being of migrants. Therefore, the aims of this study were to examine the effect of *hukou* conversion on psychological integration of rural older migrants, while also exploring the potential mediating role of accessibility of health resources.

## Methods

### Sample and procedure

The data utilized in this study are derived from the 2017 China Migrants Dynamic Survey (CMDS), a large-scale national survey on the mobile population initiated by the National Health Commission of China. The survey subjects are migrants who have resided in the destination area for one month or more. Encompassing over 340 urban agglomerations across 31 provinces in China, the survey sampled a total of 169,989 migrants in 2017. Rigorous quality control measures were applied, employing a stratified, multi-stage, and proportionally scaled probability proportional to size (PPS) sampling method to ensure the representativeness of the survey samples at both the national and provincial levels. The survey covered fundamental aspects of migrants, including basic demographics, employment and social security, health and public services, and social integration. This comprehensive data set facilitates the exploration of factors influencing and ultimate outcomes related to the psychological integration of rural older migrants.

**Fig. 1:**
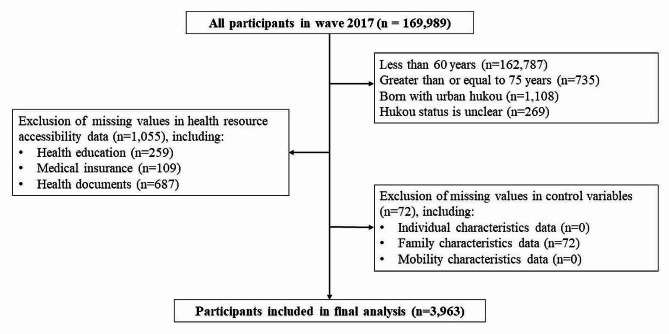
The sample selection process

Data processing includes the following procedure. Firstly, considering the age heterogeneity of older population [27], our study focused on younger older people aged 60 to 74, leading to the exclusion of 163,522 samples outside this age range. A total of 1,108 samples from the “Perpetual urban group”, those born in urban areas with urban *hukou* and remained so (urban-urban born) [22], were excluded from this study, as well as 269 samples with unclear *hukou* status. Subsequently, we removed 1,055 cases with missing values in health resource accessibility and 72 cases with missing values for control variables. Additionally, to mitigate the potential impact of outliers on study outcomes, we applied a 1% two-tailed winsorizing procedure to the household income variable. The final analysis was conducted on 3,963 valid samples, and the sample selection process is illustrated in Fig. 1.

### Variables

#### Hukou conversion

*Hukou* conversion refers to the process in China where rural migrants transfer their rural *hukou* to urban *hukou*. This study measures *hukou* conversion by taking into account both the *hukou* type and the means of acquiring the urban *hukou*. Referring to a recent study, *hukou* conversion was treated as a multi-categorical variable, which contains the following categories: (1) no conversion, where rural migrants did not convert their rural *hukou*, served as a reference group; (2) merit-based conversion, those acquired urban *hukou* through further education, joining the army, working, gaining cadre status, or buying a house; (3) family-based conversion, those acquired urban *hukou* through family ties, usually as a dependent spouse or child; (4) policy-based conversion, those acquired through land requisition or *hukou* reform; (5) other conversion, those acquired through other means [24]. While merit- and family-based conversion may exhibit higher selectivity due to personal characteristics and achievements, policy-based conversion is non-selective which is driven by policy changes.

#### Psychological integration

Building upon prior research, we employed the psychological integration scale from the CMDS questionnaire to measure the dependent variable [7, 28, 29]. This scale includes items such as “liking the current city of residence (X1)”, “paying attention to changes in the current city of residence (X2)”, “willingness to integrate into the local community and become a part of it (X3)”, “local residents’ willingness to accept me as one of them (X4)”, “attachment to customs and habits of my hometown (X5)”, “feeling that local residents look down on outsiders (X6)”, “significant differences in hygiene habits between me and local residents (X7)”, and “feeling that I am already a local resident (X8)”. Each item is categorized into four levels based on the degree of agreement: strongly disagree = 1, disagree = 2, agree to some extent = 3, strongly agree = 4.

Principal Component Analysis (PCA) was utilized to construct the main factors of psychological integration. Initially, each indicator underwent standardization. The KMO of psychological integration is 0.8327, and *P* values of the Bartlett test of sphericity are 0.000. With all eight indicators having KMO values exceeding 0.7, meeting the criterion for reasonable data structure, three principal components were ultimately extracted, contributing to a cumulative variance of 69.51%. Finally, the weighted sum of the variance contributions of each main factor, based on the ratio of each main factor’s variance contribution to the cumulative variance contribution, was calculated as the comprehensive score for psychological integration.

#### Accessibility of health resources

The mediating variable is the accessibility of health resources. Health documents, which collect and record individual disease and treatment histories, and health education, which aims to provide health knowledge and foster health awareness, are both regarded as crucial health resources [30]. Urban basic medical insurance provides insured individuals with a higher level of medical security, including reimbursement and payment of basic medical expenses, and is also considered a part of health resources [31]. This study selected three indicators as proxy variables for the accessibility of health resources for rural older migrants. These indicators include “whether a health document has been established locally”, “in the past year, whether you have received health education in the current city of residence”, and “whether you participate in basic medical insurance for urban employees or urban residents”. All the mentioned indicators are binary variables (0–1), where 1 indicates access to the respective health resources, and 0 indicates no access. On this basis, the paper constructed health resource accessibility as the dependent variable, calculated by summing up the actual obtained health project indicators. The score ranges from 0 to 3, with higher scores indicating better accessibility to health resources.

#### Covariates

Individual characteristics, family characteristics, and migration features may affect both the *hukou* conversion and psychological integration among rural older migrants [32–35]. Therefore, this study controlled for covariates in these three domains. Individual characteristics primarily include gender (0 = female; 1 = male), age (continuous variable from 60 to 74), years of schooling (educational level converted to years: never attended school = 0; primary school = 6; junior high school = 9; high school/technical school = 12; bachelor’s degree = 16; master’s degree = 20), party membership status (0 = others; 1 = CPC member), and marital status (1 = with spouse; 0 = without spouse); family characteristics include whether living with offspring (0 = no; 1 = yes) and average monthly household income (convert the total household income of continuous variables into natural logarithm); migration features include migration range (1 = cross-provincial; 2 = cross-city; 3 = cross-county), duration of migration (continuous variable of migration years), and number of migrated cities (continuous variable of migrated cities).

### Statistical analysis

Data description and analysis were performed using Stata 17. Mean, standard deviation and percentages were used to present the characteristics of the study participants. The association between *hukou* conversion and psychological integration was examined by a multiple linear regression model. To further address for selection into *hukou* conversion, a doubly robust inverse probability-weighted regression adjustment (IPWRA) method was employed [24]. Next, three regression models were conducted to test the mediating effect of the accessibility of health resources between *hukou* conversion and psychological integration. The bootstrap method with the SPSS PROCESS Macro (model 4) was used to estimate the 95% confidence interval for the mediating effect by sampling 5000 times. The mediating effect was considered statistically significant if the 95% bootstrap CI did not contain 0.

## Results

### Characteristics of study participants

Participants’ characteristics are presented in Table 1. The average psychological integration score of the participants was − 0.01 ± 1.16 (ranged − 6.01–2.09); except for 2,968 (74.89%) of participants who did not convert their rural *hukou*, 538 (13.58%) were merit-based converters, 143 (3.61%) were family-based converters, 165 (4.16%) were policy-based converters, and 149 (3.76%) were other converters; 2,363 (59.63%) were male; the average age was 64.89 ± 3.82 (ranged 60–74); Most people were with spouse (87.33%, *n* = 3,461), and few were members of the Communist Party of China (11.20%, *n* = 444); the average years of schooling were 7.08 ± 4.05 (ranged 0–19); approximately 43.55% of the participants (*n* = 1,726) were cross-provincial migrants; the average log household income was 8.25 ± 0.87 (ranged 4.09–10.31); the average number of migrated cities and duration of migration were 1.53 ± 1.70 and 10.47 ± 8.00 respectively; 1,089 (27.48%) of the participants were living with their offspring; the average score of health resources accessibility was 1.24 ± 0.94 (ranged 0–3).

**Table 1:** Characteristics of participants (*N* = 3,963)

Variables	Category	*N* (%)	M ± SD
Psychological integration			-0.01 ± 1.16
*Hukou* conversion	No conversion	2,968 (74.89)	
	Merit-based conversion	538 (13.58)	
	Family-based conversion	143 (3.61)	
	Policy-based conversion	165 (4.16)	
	Other conversion	149 (3.76)	
Gender	Male	2,363 (59.63)	
	Female	1,600 (40.37)	
Age			64.89 ± 3.82
Marital status	With spouse	3,461 (87.33)	
	Without spouse	502 (12.67)	
Years of schooling			7.08 ± 4.05
Migration range	Cross-provincial	1,726 (43.55)	
	Cross-city	1,383 (34.90)	
	Cross-county	854 (21.55)	
Political status	CPC Member	444 (11.20)	
	Others	3,519 (88.80)	
Household income (ln)			8.25 ± 0.87
Number of migrated cities			1.53 ± 1.70
Duration of migration			10.47 ± 8.00
Living with offspring	Yes	1,089 (27.48)	
	No	2,874 (72.52)	
Accessibility of health resources			1.24 ± 0.94

### Association between *hukou* conversion and psychological integration

The results of OLS and IPWRA estimators are presented in Table 2. The results of the OLS model indicate that, compared to rural migrants who retain their rural *hukou*, merit-based ($\beta$= 0.384, 95% CI: 0.265 to 0.504), family-based ($\beta$= 0.371, 95% CI: 0.178 to 0.565) and policy-based ($\beta$= 0.306, 95% CI: 0.124 to 0.487) converters have significantly higher psychological integration. However, other conversion is not significantly associated with psychological integration at the 5% level. These results remain robust even after addressing the endogenous selection bias issue using the IPWRA method. The results of the IPWRA model show that the coefficients of highly selective merit- and family-based conversion have decreased (0.384 vs. 0.353, 0.371 vs. 0.300, respectively), while the coefficient of non-selective policy-based conversion remains in line with the OLS model (0.306 vs. 0.305). This suggests that the psychological integration benefits of *hukou* conversion would be overestimated if selection bias is not accounted for.

**Table 2:** Association between hukou conversion and psychological integration (OLS and IPWRA)

	OLS			IPWRA	
	Estimate	95% CI		Estimate	95% CI
Merit-based conversion	0.384^***^	(0.265; 0.504)		0.353^***^	(0.179; 0.527)
Family-based conversion	0.371^***^	(0.178; 0.565)		0.300^*^	(0.062; 0.538)
Policy-based conversion	0.306^***^	(0.124; 0.487)		0.305^**^	(0.092; 0.517)
Other conversion	0.165^†^	(-0.025; 0.356)		0.128	(-0.055; 0.312)
Covariates	Controlled			Controlled	
Observations	3,963			3,963	

Note: †, *, **, *** respectively indicates significance at the 0.1, 0.05, 0.01, 0.001 level

The reference group is rural migrants with rural *hukou*

The coefficients are unstandardized

### Accessibility of health resources as a mediator between *hukou* conversion and psychological integration

The results of the mediation analysis and bootstrap mediating effect test are presented in Tables 3 and 4. It showed that all types of *hukou* conversion are positively associated with accessibility of health resources. After accounting for accessibility of health resources, the direct prediction effects of merit- and family-based conversion on psychological integration are significant (merit-based conversion: $\beta$= 0.230, 95% CI: 0.108 to 0.353; family-based conversion: $\beta$= 0.230, 95% CI: 0.036 to 0.423). The coefficient estimation results of policy-based conversion are not significant at the 5% level, indicating that hukou conversion in this subgroup does not have a direct impact on psychological integration. Similarly, for the subgroup of other conversions, we also did not find significant direct effects. In addition, the upper and lower limits of the bootstrap 95% interval for the indirect effects of all types of *hukou* conversion on psychological integration did not include 0 (see Table 4). This suggests that *hukou* conversion can indirectly affect psychological integration through the mediating effect of health resources accessibility.

**Table 3:** The mediating role of accessibility of health resources between *hukou* conversion and psychological integration

Variable	Psychological integration			Accessibility of health resources			Psychological integration	
	Estimate	95% CI		Estimate	95% CI		Estimate	95% CI
*Hukou* conversion (reference: No conversion)								
Merit-based conversion	0.384^***^	(0.265; 0.504)		0.783^***^	(0.691; 0.875)		0.230^***^	(0.108; 0.353)
Family-based conversion	0.371^***^	(0.178; 0.565)		0.720^***^	(0.571; 0.868)		0.230^*^	(0.036; 0.423)
Policy-based conversion	0.306^***^	(0.124; 0.487)		0.697^***^	(0.558; 0.836)		0.168^†^	(-0.014; 0.350)
Other conversion	0.165^†^	(-0.025; 0.356)		0.555^***^	(0.410; 0.701)		0.056	(-0.134; 0.245)
Accessibility of health resources							0.197^***^	(0.157; 0.237)
Gender								
Men (reference: women)	-0.140^***^	(-0.218; -0.063)		-0.021	(-0.080; 0.038)		-0.136^***^	(-0.213; -0.060)
Age	0.019^***^	(0.010; 0.029)		0.009^*^	(0.002; 0.017)		0.017^***^	(0.007; 0.026)
Marital status								
With spouse (reference: without spouse)	0.011	(-0.096; 0.119)		0.071^†^	(-0.012; 0.153)		-0.003	(-0.109; 0.104)
Years of schooling	0.020^***^	(0.010; 0.030)		0.013^***^	(0.005; 0.021)		0.017^***^	(0.007; 0.027)
Migration range (reference: cross-provincial)								
Cross-city	0.073^†^	(-0.007; 0.153)		0.148^***^	(0.087; 0.210)		0.044	(-0.036; 0.123)
Cross-county	0.160^***^	(0.066; 0.254)		0.262^***^	(0.190; 0.334)		0.108^*^	(0.015; 0.202)
Political status								
CPC member (reference: others)	0.163^**^	(0.041; 0.285)		0.116^*^	(0.023; 0.210)		0.140^*^	(0.020; 0.261)
Household income (ln)	-0.047^*^	(-0.094; -0.000)		0.006	(-0.030; 0.042)		-0.048^*^	(-0.094; -0.002)
Number of migrated cities	-0.009	(-0.030; 0.012)		0.000	(-0.016; 0.016)		-0.009	(-0.030; 0.011)
Duration of migration	0.020^***^	(0.016; 0.025)		0.005^**^	(0.001; 0.008)		0.020^***^	(0.015; 0.024)
Living with offspring	0.002	(-0.083; 0.088)		-0.030	(-0.095; 0.035)		0.008	(-0.076; 0.093)
Constant	-1.295^***^	(-2.066; -0.524)		0.097	(-0.494; 0.688)		-1.314^***^	(-2.077; -0.552)
Observations	3,963			3,963			3,963	
R²	0.064			0.166			0.086	

Note: †, *, **, *** respectively indicates significance at the 0.1, 0.05, 0.01, 0.001 level

The coefficients are unstandardized

**Table 4:** Bootstrap test for mediating effect

	Effect	Estimate	95% Bootstrap CI
Merit-based conversion	Total effect	0.384^***^	(0.265; 0.504)
	Direct effect	0.230^***^	(0.108; 0.353)
	Indirect effect	0.154^***^	(0.118; 0.192)
Family-based conversion	Total effect	0.371^***^	(0.178; 0.565)
	Direct effect	0.230^*^	(0.036; 0.423)
	Indirect effect	0.142^***^	(0.100; 0.189)
Policy-based conversion	Total effect	0.306^***^	(0.124; 0.487)
	Direct effect	0.168^†^	(-0.014; 0.350)
	Indirect effect	0.137^***^	(0.099; 0.182)
Other conversion	Total effect	0.165^†^	(-0.025; 0.356)
	Direct effect	0.056	(-0.134; 0.245)
	Indirect effect	0.110^***^	(0.077; 0.150)

Note: †, *, **, *** respectively indicates significance at the 0.1, 0.05, 0.01, 0.001 level

The coefficients are unstandardized

## Discussion

This study investigates the underlying mechanisms of *hukou* conversion on psychological integration from the perspective of a representative sample of rural older migrants in China. The results indicate that *hukou* conversion positively predicts psychological integration, and the accessibility of healthcare resources plays a significant mediating role in this process. These findings contribute to a deeper understanding of the influencing factors of psychological integration among rural older migrants, thereby contributing to the improvement of people’s well-being and the establishment of mechanisms for integrated urban-rural development.

To start with, our study extends the literature on the impact of the *hukou* system on psychological integration among rural older migrants. First, existing literature predominantly focused on discussing the effects of the static *hukou* system. A recent study pointed out that having urban *hukou* makes it easier to access more public services and social welfare compared to rural *hukou* [36]. Another study, based on empirical data from 1,100 rural migrants, found that the *hukou* system perpetuates the long-term marginalization of rural migrants, leading to continuous barriers in social/cultural and identity integration [37]. However, little is known about the health impacts of dynamic *hukou* conversion currently. This study contributes to this gap by estimating the health impacts of dynamic *hukou* conversion for rural-to-urban migration. Second, existing literature primarily focused on migrant adolescents and workers rather than the older population. With increasing age, older adults tend to invest more in their original social networks, resulting in higher psychological integration costs associated with migration [38], making this group both important and unique. Our study further focused on the older adults, aged 60 to 74, showing that conversion from rural *hukou* to urban *hukou* significantly enhances the psychological integration level of older migrants compared to those who have consistently held rural *hukou*, aligning with the conclusions drawn in existing literature [39, 40].

Moreover, this study classified the “rural to urban” population based on different ways of obtaining urban *hukou*. We found that merit-based, family-based, and policy-based conversions all lead to higher levels of psychological integration. The health benefits of merit- and policy-based conversion are consistent with the findings of existing research focusing on psychological well-being [24]. Since policy-based conversion is theoretically the least selective type of *hukou* conversion, its health benefits show almost no difference after correcting for selection bias using the IPWRA method, compared with a significant difference of the more selective merit- and family-based conversion. A previous study stated that merit- and family-based converters experienced a migration process, which may introduce a variety of risk factors for individual well-being, such as the stress associated with adapting to a new physical and social environment, while policy-based converters do not involve a migration process [24]. Hence their findings demonstrated that policy-based conversion had the largest health benefits, which is inconsistent with our findings. However, another study confirmed the advantages of selectivity, stating that highly selected *hukou* converters might have the best outcomes because this subset tends to comprise individuals who excel among those with rural *hukou*, often having achieved higher levels of schooling and be healthier [41]. Our findings showed that the health benefits of highly selected merit- and family-based conversion are greater than policy-based conversion. Since we focused on psychological integration, we believed that merit- and family-based converters, who have higher human and social capital, are more proactive in *hukou* conversion, and they will enhance their sense of identity and psychological integration in this process.

Furthermore, our study found that *hukou* conversion significantly improves the accessibility of health resources, while the accessibility of health resources also promotes the psychological integration of rural older migrants. On the one hand, the urban-rural gap in China is an important institutional background [42]. The inequality in public welfare and health care caused by strict household registration restrictions has been widely confirmed [14, 43], with only those holding local urban *hukou* being able to access more generous social insurance [44]. A recent study found that lifting barriers to local citizenship significantly increases the participation rate of migrant workers in medical services, significantly improving the accessibility of welfare benefits and public services [39]. This study confirms and extends this conclusion, showing that merit-based, family-based, and policy-based conversion all significantly enhance the accessibility of health resources. On the other hand, previous study found that immigrants’ inability to access healthcare services equally not only affects physical health but also leads to negative mental health outcomes [45]. Previous scholars have found that health insurance can promote the social integration of migrants and have discussed the positive correlation between the social integration of migrants and the utilization of basic public health services in a broad scope (all migrants above 15 years old) [46, 47]. Our study takes a more comprehensive approach to assess the accessibility of health resources and focuses on the rural older migrants. We confirm that the attainment of accessibility to health resources significantly predicts psychological integration positively, providing insights for safeguarding the quality of life for rural older adults.

The results of the Bootstrap analysis suggest that the indirect effect mediated by accessibility of health resources was significant between all types of *hukou* conversion and psychological integration. Compared with individuals holding rural *hukou*, *hukou* converters have pronounced advantages in accessing healthcare throughout their life, shaping better health outcomes [22]. In addition, the direct effect of policy-based conversion on psychological integration is not significant at the 5% level, suggesting that accessibility of health resources has a full mediation effect on the relationship between policy-based conversion and psychological integration. Since policy-based conversion is due to the incorporation of villages into urban areas [41], they can enjoy urban services by acquiring urban *hukou* but do not experience a migration process. Therefore, *hukou* conversion may not directly increase their psychological integration, only through the benefits of healthcare access.

### Limitation

This study utilized cross-sectional data from the 2017 CMDS. Limited by the availability of research data, we were unable to observe the long-term effects of *hukou* conversion on the psychological integration of rural older migrants, including the sustainability of effects, trends in changes, and potential stage differences. Therefore, future research could consider employing longitudinal data to further explore the long-term impact mechanisms of *hukou* conversion on the psychological integration level of rural older migrants. Moreover, since mediation consists of causal processes that unfold over time, the cross-sectional design may generate biased estimates. Future studies are required to employ alternative longitudinal mediation models to establish robust causality. Finally, although we attempted to address the selection bias of *hukou* conversion using the IPWRA method, this approach also has limitations, including the correct simulation of all factors involved in selective entry analysis. While we have included a large number of covariates in our model, we cannot completely eliminate the possibility of unobserved confounders. Further research is needed to seek additional validation and confirmation in the future.

## Conclusion

The results of this study demonstrate a significant correlation between *hukou* conversion, accessibility to healthcare resources, and the psychological integration of rural older migrants. Accessibility to healthcare resources plays a mediating role in the impact of *hukou* conversion on psychological integration. These findings hold important implications for improving the psychological integration level of rural older migrants and promoting social harmony and stability.

*Hukou* conversion has a crucial impact on the social adaptability and psychological well-being of rural older migrants, underscoring its significance in fostering their psychological integration. It is imperative to strengthen the implementation of *hukou* reform policies, establish a unified *hukou* system between urban and rural areas, and provide equalized public services and welfare. Policymakers should focus on enhancing the healthcare rights protection system for rural older migrants, improving the accessibility of healthcare resources. Efforts should be made to ensure that older migrants in destination areas enjoy the same benefits as local residents, thereby promoting deeper psychological integration among rural older migrants.

## Data Availability

The datasets generated and/or analysed during the current study are available in the CMDS repository, https://www.chinaldrk.org.cn/wjw/#/data/classify/population/yearList. The webpage requires registration to access the data. You can register an account yourself and download the data through public channels. Alternatively, you can directly contact the author Tianxin Cai (51254403013@stu.ecnu.edu.cn), and we would be happy to provide you with the original data for academic exchange.
